# Supplementary material for: Are carbon emissions trading and green financial instruments synergistic? -Comprehensive quantitative research based on content analysis
Source: PLoS One. 2024 Mar 7;19(3):e0298601. doi: 10.1371/journal.pone.0298601 (PMC10919601; doi:10.1371/journal.pone.0298601)
Supplement: S1 Data — (ZIP) [file pone.0298601.s001.zip › Data/地级市Z_F工作报告.zip!/╣π╢1⁄2/╜╥╤⌠╩╨/└┤╫╘╣1⁄2╓┌║┼í╛┬φ┐╦╩2╛▌═°í┐_2017.docx]

各位代表：

现在，我代表市人民政府向大会作工作报告，请予审议，并请市政协各位委员和其他列席人员提出意见。

过去五年工作回顾

过去五年，我们在省委省政府和市委的坚强领导下，在市人大及其常委会和市政协的监督支持下，坚持以党的十八大和十八届三中、四中、五中、六中全会精神为指导，深入学习贯彻习近平总书记系列重要讲话精神，牢固树立五大发展理念，主动适应经济发展新常态，扎实推进供给侧结构性改革，围绕“三个定位、两个率先”目标，全面落实省促进粤东西北地区振兴发展和创新驱动发展两大战略，按照市委“打造粤东发展极，建设幸福新揭阳”的发展目标和“抓落实、促发展，抓基层、强基础”的工作部署，坚持“强化效率、彰显公平、惠民执政、绿色发展”的施政理念，改革创新，攻坚克难，锐意进取，全力推动经济社会发展，较好地完成了“十二五”规划的目标任务，实现了“十三五”的良好开局。

——五年来，我们主动适应经济发展新常态，全面深化供给侧结构性改革，努力促进经济持续健康发展。2016年，预计全市生产总值2012亿元，五年年均增长10.1%；规模以上工业增加值1135亿元，年均增长14.7%；固定资产投资1478亿元，年均增长24.4%；社会消费品零售总额977亿元，年均增长14.3%；外贸进出口463.8亿元，年均增长10.9%；地方公共预算收入77.4亿元，年均增长10.8%；城乡居民人均可支配收入17560元，年均增长11.2%；金融各项存款余额2015亿元，贷款余额986亿元，分别比2011年底增长78.3%和96.4%。

——五年来，我们抢抓省实施促进粤东西北地区振兴发展战略契机，全力加快交通基础设施、产业园区和中心城区扩容提质“三大建设”，着力夯实经济社会发展基础。交通基础设施建设五年累计投资223.67亿元，揭阳潮汕国际机场成为广东省第三个国际机场；惠来2个10万吨级泊位竣工使用，大南海公共码头前期工作扎实推进；厦深高铁运行，梅汕客专揭阳段开工；潮惠高速揭阳段、汕湛高速揭博段通车，实现“县县通高速”；改造国、省道277公里，新改建地方公路2180公里。中德金属生态城成为华南地区首个中德中小企业合作区；揭阳产业转移园等6个园区纳入省产业转移园区和产业集聚区，五年累计投入园区建设411.63亿元；空港经济区、大南海石化工业区等主题产业平台规划建设。中心城区扩容提质步伐加快，揭东撤县设区，发行首期城投债16亿元，获省粤东西北股权基金9.3亿元投入新区建设、成功申请国家政策性融资15亿元启动市区棚户区改造，争取国开行、农发行专项建设基金11亿元。建设市区儿童公园、贝多芬生态公园首期以及晓翠路北段、临江南路改造等一批市政项目。开展文明创建活动，环境卫生和交通秩序专项整治取得成效，市容市貌明显改观。

——五年来，我们认真落实创新驱动战略，实施产业转型升级“6166行动计划”和“产业引进”“产业登高”两大工程，持续推进产业强市建设。加强产业组织化建设，成立金属、服装、玩具、电线电缆、电子商务等一批行业商协会。中委广东石化炼化项目落地建设，中海油LNG项目竣工，惠来电厂1-4号机组投产。实施工业转型升级攻坚战行动，每年保障50%以上土地指标用于工业项目，完成工业技改投资793亿元。2016年，预计金属、纺织服装、石化、医药、食品五大产业增加值752亿元，比2011年增长112.5%。创建省金融科技产业融合创新综合试验区，揭东、普宁农信社改制为农村商业银行，新增上市公司1家、新三板挂牌企业4家，新增省级工程中心25个、科技企业孵化器6家，设立院士工作站8个，高新技术企业达51家。举办中德（欧）购物节、玉文化节、衬衣节、电商年货节等系列展会。新增国家4A旅游景区3家、四星级旅游饭店3家，揭西县获批创建国家全域旅游示范区。

——五年来，我们依托国家“一带一路”战略，积极开展以德国为重点的国际合作，拓展对外开放新局面。创建全国首个德国工业园区参与规划设计管理营运的中德金属生态城。承办中德政党对话会和中德中小企业政策磋商会，举办中德中小企业合作交流会并升格由省政府和工信部联合主办，组建对德（欧）合作“百人团”，在德国、奥地利、西班牙、捷克、法国创建10个办事处。中德资源再生基地、德国先进技术合作中心、电镀“零排放”项目在中德两国领导人见证下签约，落户中德合作企业34家，保库智能管网、戴维特余热发电机等5个项目建成投产。扎实拓展与“一带一路”沿线国家地区的经贸合作，形成全方位开放格局。

——五年来，我们全面实施“互联网+”计划，突出抓好“一基地、两大赛、三工程”，以电商带动形成“互联网+”发展优势。实施十万电商人才培训工程，累计培训电商人才16万人。成功创建国家电子商务示范城市和工业电子商务区域试点城市，揭东锡场、揭西金和分别成为全省首批“互联网+”创建小镇和培育小镇；揭东区荣获2015年广东省县域电商创新奖。全市淘宝村达50个，列全省第3位，普宁跻身全国淘宝村集群第3位。设立中药材、油品两个大型电子交易平台，发布中国首个中药材价格指数。2016年全市快递业务量预计达4亿件，比上年增长258.2%，开通首条全货机航线，成为广东首个“国家快递示范城市”。

——五年来，我们以农业“一镇一品”工程为切入，培育形成农村经济新动能，促进“三农”提质增效。创建“一镇一品”工程示范市，建设30个重点示范镇，形成30个特色农业品牌，成功打造4个国家级“一村一品”示范镇（村）。连续5年完成省下达的粮食考评任务。开工建设神泉现代示范性渔港。投入水利建设资金51.42亿元，完成普宁白坑湖水库综合整治等52宗重点水利工程。投入农网改造资金20.48亿元，新建10千伏供电线路942.55公里。启动省级新农村连片示范工程项目181宗，落实美丽乡村“三大工程”建设项目2513宗。实施“农村清洁工程”，建成69座镇生活垃圾转运站和3410个自然村垃圾收集点。实现全市农村“三资”管理服务平台全覆盖。投入扶贫资金17.08亿元，扶持贫困村项目5177个、贫困户项目13.04万个，帮助脱贫28.47万人。2016年，预计农民人均年可支配收入12330元，年均增长11.8%。

——五年来，我们始终坚持绿色发展理念，铁腕向污染宣战，千方百计补齐环保短板。建成全国首个“零排放”电镀产业园，榕江流域基本实现无电镀污染。建成市区污水处理厂二期等9个污水处理项目，纳入“PPP”的9个污水处理厂、普宁市生活垃圾环保处理中心等项目建设进展顺利。关停非法电镀加工企业270家、土轧钢企业50家、漂染企业150家，清理整治普宁电子废物经营户942户。2016年，城市空气质量达标率92.5%；榕江11个常规监测断面总体保持稳定；练江青洋山桥断面主要污染物化学需氧量、氨氮、总磷平均浓度比2011年分别下降55.8%、45.8%、48.7%。倡导实践“人树一体”理念，新增森林碳汇造林52.99万亩，建设生态景观林带226.4公里，新增森林绿地面积8.9万亩，森林覆盖率提高至55.54%。揭西县获批国家级生态保护与建设示范区，继揭西、揭东之后，榕城、普宁、惠来获省政府授予林业生态县称号。

——五年来，我们认真践行惠民执政理念，着力保障和改善社会民生，让人民群众共享发展成果。五年教育总投入336亿元，建成标准化义务教育学校1038所、规范化幼儿园595所，改造提升薄弱普通高中22所，实现创建广东省教育强市目标，率先在粤东地区实现省教育强镇、省教育强县和国家级义务教育发展基本均衡县“三个全覆盖”，教育质量居粤东西北地区前列。投入医疗卫生资金30亿元，市慈云医院、康美医院、普宁市中医院、揭西县中医院、惠来县人民医院等一批卫生设施建成投用，创建“三甲医院”3家，医改工作取得明显成效。全市病床、卫生计生技术人员、执业（助理）医师和注册护士分别比2011年末增长77.8%、59.2%、21.9%和86.3%。生育政策改革得到落实。城乡居民基本养老保险和基本医疗保险均实现全覆盖。新增国家级文物保护单位3处，市图书馆建成开放使用，市档案馆晋升为国家二级档案馆。获得“广东省双拥模范城”荣誉称号，阳光征兵经验在全省推广。为全市人民统一购买政府民生综合责任险，为70岁以上老人投保意外伤害险，为百岁老人、本科以上贫困大学生、乡镇卫生院全日制本科学历以上医生发放津贴补助，免除公立幼儿园在园低保户儿童保教费，投放新能源公交车506辆。全面铺开“一村（社区）一法律顾问”工作，社会治安和信访维稳得到强化，生产安全、消防安全、食品安全形势保持稳定。

——五年来，我们把建设人民满意政府作为努力方向，切实转变政府职能，政府自身建设得到加强。深入开展党的群众路线教育实践活动、“三严三实”专题教育和“两学一做”学习教育，严肃整治“四风”和不严不实问题。坚持向人大及其常委会报告工作，向政协通报情况，自觉接受监督。共办理市人大代表建议117件、市政协提案345件。市、县两级政务服务中心和网上办事大厅投入使用，建成四级政务服务体系。完成新一轮政府机构改革，推进商事登记和行政审批制度改革，取消、下放行政审批事项204项，全面推行“五证合一、一照一码”登记制度改革。出台《揭阳市复制推广中国（广东）自贸区创新经验加快空港经济区发展的意见》。市政府系统议事协调机构精简49%。公务用车制度改革顺利完成。开展行政复议委员会试点工作，建立政府法律顾问制度。强化行政监察和审计监督，政府系统党风廉政建设得到加强。

过去五年，成绩来之不易。这是党中央国务院、省委省政府和市委正确领导的结果，是市人大、市政协监督支持的结果，是全市人民共同奋斗、社会各界大力支持的结果。在此，我谨代表市人民政府，向全市干部群众，向驻揭解放军和武警部队官兵，向关心支持揭阳改革发展的各界朋友、港澳台同胞、海外侨胞和国际友人表示衷心感谢并致以崇高敬意！

在看到成绩的同时，我们也清醒地认识到，当前经济社会发展仍存在不少困难和问题：经济发展方式总体粗放，产业转型升级任重道远；民生事业欠账较多，公共服务水平有待提高；环保生态短板突出，节能减排任务繁重；社会管理面临新矛盾新挑战，维护稳定压力较大；制约发展的体制机制障碍依然存在，政府职能转变亟待深化，等等。对此，我们将高度重视，采取有力措施，认真加以解决。

今后五年目标任务

今后五年是全面建成小康社会的决胜期，也是揭阳振兴发展的攻坚期。当前，世界经济在深度调整中曲折复苏，国内经济发展进入新常态，经济增长“减速换档”，新一轮科技革命方兴未艾，揭阳发展正处在加快调整的阵痛期，也面临时代变革的“机会之窗”。机遇与挑战并存，希望与困难同在，只要我们勇于担当，敢于作为，奋发进取，扎实工作，就一定能够实现揭阳跨越崛起、振兴发展的美好愿景！

今后五年政府工作的总体要求：紧密团结在以习近平总书记为核心的党中央周围，全面贯彻党的十八大和十八届三中、四中、五中、六中全会精神，深入贯彻习近平总书记系列重要讲话精神，按照“五位一体”总体布局和“四个全面”战略布局，坚持稳中求进工作总基调，适应把握引领经济发展新常态，牢固树立和积极践行创新、协调、绿色、开放、共享五大发展理念，全面落实省促进粤东西北地区振兴发展和创新驱动发展两大战略，按照市第六次党代会的部署，抓落实、促发展，抓基层、强基础，推动经济健康快速发展，确保社会大局和谐稳定，不断提高人民生活水平，如期实现全面建成小康社会的宏伟目标。

今后五年经济社会发展主要预期目标：主要经济指标增速快于全省平均水平，力争到2020年，全市人均GDP、居民人均可支配收入接近全国平均水平，把揭阳建设成为新型工业城市、生态宜居城市、和谐文明城市。

实现以上奋斗目标，必须始终做到“六个坚定不移”：

——坚定不移深化各项改革。按照中央全面深化改革的顶层设计，推进重要领域和关键环节的改革。把供给侧结构性改革作为统领经济工作的主线，扎实抓好去产能、去库存、去杠杆、降成本、补短板，重点是精准发力补齐基础设施和公共服务短板，通过简政放权降低企业成本。围绕充分发挥市场对资源配置的决定性作用和更好发挥政府作用，推进各领域综合改革，促进思想观念转变和利益格局调整，营造市场化法治化国际化的营商环境。

——坚定不移落实创新驱动战略。以对德合作为依托，高水平建设中德工业4.0合作试验区，把揭阳打造成为立足广东、面向全国的德国先进技术推广中心和德国先进装备国产化中心，全方位实现合作型创新。牢固树立互联网思维，推动“互联网+”在经济社会各领域延伸拓展，以互联网推动产业技术创新、商业模式创新、城市治理创新。高度关注人才和青年发展，大力发展众创空间，着力打造友善包容的创业创新环境，激发社会创业创新活力。

——坚定不移发展实体经济。保持定力，巩固基础，在实体经济上持续下笨功夫、实功夫。全面实施《“揭阳制造2025”发展规划》，在推进传统产业转型升级的同时，发挥中委石化炼化项目带动作用，延伸产业链条和配套服务，着力发展壮大石化产业。以电镀“零排放”为切入，深化与德国环保集群的对接，重点发展环保装备产业。面向未来培育发展装备制造、大健康等新兴产业，努力推动揭阳产业迈向中高端。以行业协会带动企业抱团发展，推动民营经济实现新一轮大发展。按照“一县一主题产业”的总体思路，深入推进产业登高、产业引进、产业共建，形成主题鲜明的县域经济格局。

——坚定不移促进城乡协调发展。加快城市基础设施建设，推动中心城区扩容提质。统筹城乡发展，均衡配置资源，力促城乡协调发展。着力促进空港、海港、河港、高铁、高速等的内外互联互通及快速转换，强化立体交通体系的可及性和辐射力。超前建设新一代信息化基础设施，打通经济社会发展的“大动脉”。以建设国家级快递物流产业园为引领，形成高效便捷通达的物流体系，更好地发挥国家快递示范城市的品牌效应和“虹吸效应”。加快以人为核心的新型城镇化，提升县城和中心镇基础设施水平，大力培育发展特色小镇，推动基础设施和公共服务向农村延伸，带动农村一二三产业融合发展。

——坚定不移推进绿色发展。牢固树立绿色发展理念，统筹抓好水、大气、土壤污染整治，修复生态环境，推广绿色制造，倡导低碳生活，形成生产、生活、生态和谐一体的发展模式。实施环保基础设施建设三年行动计划，努力补齐环保设施短板。借鉴莱茵河治理经验，提升推广电镀“零排放”模式，铲除电镀、酸洗、漂染“三大病灶”，全面消除城市建成区黑臭水体，全面提升榕江、练江水质。深入推进新一轮绿化揭阳大行动，建设绿色揭阳。

——坚定不移增进民生福祉。做大做强产业经济，为老百姓创造更多创富增收机会，确保按期完成精准脱贫目标，与全国同步全面建成小康社会。创建省教育现代化先进市和卫生强市，健全公共服务体系。尽力而为，小步快跑，每年为老百姓办成一批好事实事，完善社会保障体系，让人民有更多获得感。深入开展社会主义核心价值观教育，提升市民素质，建设文明城市。全面推进依法治市，建设法治政府，维护社会公平正义，为城市发展创造和谐稳定环境。

2017年工作安排

2017年是新一届政府工作的起始之年，也是实施“十三五”规划的重要一年。我们将深入学习贯彻习近平总书记系列重要讲话精神和治国理政新理念新思想新战略，在省委省政府和市委的领导下，坚持稳中求进工作总基调，以推进供给侧结构性改革为主线，着力稳增长、促改革、调结构、惠民生、防风险，积极推动经济平稳较快发展，切实维护社会和谐稳定，为新一届政府工作开好局、起好步。

今年经济社会发展主要预期目标：生产总值增长7%以上；固定资产投资增长11%；社会消费品零售总额增长13%；外贸进出口总额增长6%；地方公共预算收入增长5.5%；城乡居民人均可支配收入增长8%；居民消费价格涨幅控制在3%以内；城镇登记失业率控制在3.5%以内；节能减排约束性指标完成省下达年度计划。

（一）全面实施创新驱动，聚力振兴实体经济

全力以赴打造支柱产业。加快中委广东石化炼化项目建设，支持推动向炼化一体延展，扩大化工布局，拓展产业链条，探索建设炼化供给侧结构性改革基地。加快园区道路、公共码头、环保中心、公共罐区、公共管廊等基础设施建设；与上海化学工业区、中国化学工业联合会、中国石油大学等开展务实合作，实现平台共建互通共享；梳理对接潮汕籍石化产业企业，实施石化产业乡贤回归工程，建设潮汕民营化工产业聚集区。精耕细作“零排放”电镀园区，强化品牌、标准、技术及产业链效应，整合吸纳绿色电镀产业、绿色电镀科研、绿色电镀上下游产业“三聚集”，建设中德合作绿色电镀产业合作区。加快新地、德贝斯、威特普等首批11家中德（欧）先进环保装备企业落地投产，在环保产业发展的“风口期”，高效率、有规模地建成环保装备产业集群，抢占产业发展新高地。推动纺织服装、金属加工、医药食品、模具、鞋业、玩具等传统产业转型升级。实施转型升级领军企业家培育工程，努力培育一批有国际视野、有宽广胸襟、能拥抱新技术新模式的新型企业家；实施转型升级灯塔企业培育工程，集中力量，锁定目标，培育一批在技术研发、标准制定、绿色生产、智能生产、现代营销、现代化管理等方面先行先试的示范企业；实施转型升级公共服务平台建设工程，以协会、园区或产业集群为单位，集中资源，支持建设一批大数据、研发设计、线上线下一体化营销、仓储物流、双元制培训及产业金融等产业公共平台，推动企业落子布局产业双高端，再造传统产业新优势。

持之以恒提升质量品牌。常态化开展全市性的质量大竞赛活动，引导企业树立质量第一的强烈意识和目标追求，抱团打造产业联盟标准，制定落实惩劣奖优行业规约，严惩假冒伪劣的“害群之马”，努力把揭阳建设成为实体经济中的质量明星。加强知识产权司法和行政协同保护。强化品牌建设，打造“百年老店”。鼓励支持行业协会、产业集群开展产业品牌培育活动，以企业品牌的集聚，实现产业品牌的扩张。放大“中德中小企业合作区”“大南海石化工业区”“中国五金基地市”“中国纺织服装基地市”“中国塑料时尚鞋之都”“中国中药名城”“国家电子商务示范城市”“国家快递示范城市”“中国玉都”“军埔电商村”等区域性品牌的影响力、竞争力，集聚技术、营销、人力、金融等更多资源要素，打造要素齐全、链条完善的品牌产业。

内外联动推进产业共建。用足用好用活省产业共建扶持政策，把握机遇窗口，以县（市、区）为单位，建立产业共建领导机构，出台产业共建政策，确定产业共建目标，规划建设产业共建平台，改善产业共建的硬软件服务配套，开展产业共建竞赛活动，以产业共建形成县域经济增量，形成与珠三角形优势互补的区域产业合作分工新格局。大力实施“乡贤回归”工程，积极动员在外潮籍企业家投身揭阳产业共建。

脚踏实地发展县域经济。坚持“一县一主题产业、一产业平台、一产业链条、一产业会展、一产业政策、一产业文化”的发展方向，精准发力，久久为功，挖掘县域资源禀赋，把握产业发展规律，顺应产业发展脉络，找准产业建设关键环节，脚踏实地推进产业建设。榕城区重点梳理街区经济，加大教育、文化、医疗、餐饮产业的组织整合提升力度，抓好后汽车产业及鞋业创意产业，推进双创平台、孵化器等产业空间建设，发展动漫、网游等新兴产业。揭东区依托中德金属生态城和军埔电商村，探索先进制造业与“互联网+”协同发展的路子，加快揭东经济开发区转型升级，加快“四站合一”高铁站园一体化规划建设，以平台优势带动形成产业优势。产业转移园以玉产业转型升级启动文化产业构建，以海峡食品药品合作区、高端装备制造及电子商务小微企业加工聚集区建设实现产业聚集。空港经济区借助机场扩容、航空业务拓展的契机，下大力气建设国家级快递物流产业园，开发培育大数据产业、临空制造业，推进模具、玩具、家具、石材产业转型升级，以空港优势带动形成物流优势、产业优势。普宁市着力建设纺织服装生态园区，以绿色环保要素铸造纺织产业竞争力；以资本要素引导人才、科技要素聚集，发展大健康产业；着力发展电子商务，建设互联网+商贸高地。揭西县坚持生态发展，发展互联网+生态旅游、生态农业和新型电线电缆产业。惠来县坚持海洋经济、滨海制造业、滨海旅游产业、农海产品加工生产并举，形成临海产业格局。大南海石化工业区争取成为石化供给侧改革试验区。普侨区、大南山侨区主动与普宁、惠来、大南海对接，发展生产性服务业、绿色生态农业、健康养老产业。

凝心聚力发展民营经济。加强行业协会建设，推进民营经济集群发展。深入开展“法律、科技、金融、人力资源、物流”五大暖企行动，为民营企业发展加油减压、清障护航，引导集聚民间资本发展实体经济。加强民营经济服务平台和服务体系建设，推进“个转企、小升规、规转高”的民营企业培育计划，重点培育一批高新技术企业，培育一批上市公司，培育一批产值超10亿元以上的大中型骨干企业，培育一批风投、创投企业。

悉心呵护培育新兴业态。继续抓好“一基地、两大赛、三工程”，建设面向全国的电商免费培训基地，办好中国电商好讲师邀请大赛和中国电商人才擂台大赛，实施电商下乡、电商进厂、跨境电商建设工程。推动传统产业与电商对接互动，为传统产业插上“互联网+”的翅膀。加快揭东锡场、揭西金和“互联网+”小镇建设，推动互联网+服装、互联网+玩具、互联网+旅游试点工作，探索建设电商产品标准化体系，强化电商产业诚信建设，建设名副其实的国家级电子商务示范城市。

（二）扎实推进对德合作，构筑对外开放新格局

全力推进项目落地扎根。加快戴维特余热发电机、保库智能管网等项目达产运营；加快巨轮工业机器人、新地环保装备等在建项目建设投产；加快凯斯宝玛、威特普等在谈项目落地建设，努力实现百家中德（欧）先进企业聚集落地的目标。建设中德电镀研究院、中德环保装备研发中心、中德先进技术引进中心。提升办好对德合作“百人团”，落实“引进一名德国退休工程师，引进一位德国合作伙伴，引进一套德国企业文化和管理制度，引进一套德国先进技术，引进一套德国先进设备”的“五引进”目标，实现“百人团”企业转型升级、示范引领；举办对德合作青年精英培训班，培育一批懂德国制造业文化、有德国合作资源、畅通对德合作路径、担当对德合作先锋的青年企业家队伍；办好市民德语夜校，夯实对德合作发展实体经济的群众基础，实现以人为重点的对德制造业合作。

努力促进外贸转型升级。借鉴自贸区创新经验，建设产业国际合作服务平台，推广金属产业国际合作经验，引导纺织服装、医药食品、模具、玩具、鞋业等产业强化国际合作。加快建设跨境电子商务园区，申报海关特殊监管区，完善“公共服务平台+集中监管区”监管新模式和退税、跨境支付、物流等支撑系统，引导企业以“互联网+一般贸易”模式，通过海外仓和海外运营中心融入境外零售体系。着力培育外贸综合服务企业，为中小微企业出口提供集成服务，打造外贸转型升级示范基地和公共服务平台，建设境外营销服务体系。推动建设加工贸易转移基地，调整优化外贸结构，培育打造出口品牌，开拓多元化市场。

大力提升对外开放水平。加快完善中德金属生态城园区基础设施建设，重点建设中德中小企业家合作培训基地、中德合作学校、中德合作医院及贝多芬生态公园。进一步发挥驻德（欧）办事机构的作用，承建德国“中国中心”，办好第三届中德中小企业合作交流会，加快营运中德跨境电商产业园，提升中德（欧）购物节办节水平，把揭阳建设成为中德合作重要平台。发挥海外侨胞的桥梁纽带作用，积极拓展国际友城，加强与“一带一路”国家的经贸合作，努力形成以对德合作为重点，有效对接欧美发达国家及“一带一路”沿线国家的全方位对外开放新格局。

（三）坚持规划建设并举，夯实城市发展基础

加快立体交通体系建设。启动揭阳潮汕国际机场扩建；加快梅汕客专揭阳段建设；抓紧上马大南海公共码头、揭阳疏港铁路项目，谋划建设榕江港口群；加快揭惠、汕湛高速揭阳段建设步伐，开工建设汕尾至瑞金高速揭阳段，推进揭惠高速市区连接线建设，推动国道省道改造升级，规划建设揭东至揭西快线，统筹地方道路建设。科学规划建设空港换乘中心和揭东“四站合一”平台，把揭阳建设成为空港、海港、河港、高铁、高速“五位一体”的交通枢纽城市。加强快递物流平台建设，打造国家快递示范城市金字招牌。

提升城乡建设管理水平。完成《揭阳市总体规划（2015-2030）》修编和全市土地利用总体规划调整完善工作，按照“三规合一”“两图合一”的原则，抓好城市总体规划与市政道路、地下管廊、公共服务体系、生态环保等专项规划修编衔接。突出规划建设好“市肺”“城市氧吧”、一河两岸、依山傍水、历史文脉、城市门户、“城市客厅”等重点板块。建设空港-金属城连接线、临江北路西段等13条市政道路；规划启动新城大桥、莲花大桥、榕江景观大桥；加快黄岐山森林公园、贝多芬生态公园、临空农业公园、“两河四岸”绿色长廊建设及古城保护开发。新建扩改升级一批教育、文化、体育、医疗等公共服务项目。严格执行城乡规划，依法管治非法建筑、小产权房，理顺“三旧”改造遗留问题，加快棚户区改造项目建设。完善城市管理体制，提升城市管理水平。在环境卫生、园林管理等领域，实施政府购买社会服务，激发管理活力。通过“PPP”等方式，吸引社会资本参与城市基础设施建设，加快城市发展步伐。

优化拓展城镇发展布局。以更高的站位布局全市城镇发展，在“一带一路”中彰显中德中小企业合作区的国际合作地位；在临港空铁合作区中彰显空港经济区的交通枢纽地位；在潮汕文化传承中挖掘古城的文脉功用；在“互联网+”建设中突出军埔电商的引领作用。以交通连接、产业对接、文化融合促进城乡一体化建设，以县城承接力的拓展、县域创富能力的提升，以特色专业小镇的规划建设、特色专业小镇的错位互补发展，实现揭阳协调共享发展。今年各县（市、区）重点推进县、镇医疗、教育及环保基础设施建设，重点谋划建设有可持续资源支撑的特色小镇，在县城建设中突出生产、生活、生态“三生一体”，以城镇化建设提升人民群众的获得感。

（四）抓好农业供给侧改革，促进“三农”提质增效

推进“一镇一品”建设。把增加绿色优质农产品供给放在突出位置，以30个“一镇一品”示范镇为引领，狠抓农产品标准化生产、品牌创建、质量安全监管，让人民群众吃上绿色、安全、放心食品。加快培育新型经营主体，推进经营主体接二产连三产，延长产业链条，提高产业附加值。结合发展“全域旅游”，整合“一镇一品”、生态旅游、健康养老、文化资源等多领域元素，加强项目设计，大力发展观光农业、休闲渔业、田园城市，形成农村经济新亮点、农民增收新抓手。

加强农村基础建设。落实最严格耕地保护制度，全面完成永久基本农田划定工作，改造和建设一批高标准农田，扩大“沃土工程”实施规模，稳定粮食产能。加快邦山水闸重建、惠来县葫芦潭水库除险加固、惠来神泉现代示范性渔港等重点工程建设，加大农网改造力度，推进互联网基础设施进村入户，夯实农村发展新基础。开展农村人居环境整治行动，加强农村垃圾和污水收集处理设施以及防洪排涝设施建设，高标准推进美丽乡村“三大工程”。

大力实施精准扶贫。落实各级党政一把手抓扶贫责任制，按照转移就业脱贫一批、发展生产脱贫一批、生态补偿脱贫一批、发展教育脱贫一批、社会保障兜底一批的要求，全面深化精准扶贫工作。重点规划建设揭西县金和镇“互联网+扶贫小镇”，建设绿色农产品采购、加工、仓储物流冷链、电子商务、质量检验检测五大中心，打造实施“互联网+扶贫”战略的支点项目，统筹推进全市精准扶贫、精准脱贫。落实农村危房改造任务，解决贫困群众住有所居问题。

（五）推进关键领域改革，增强揭阳发展活力

深化供给侧结构性改革。按照中央和省的部署要求，全面落实“三去一降一补”行动方案，重点在“降”和“补”上下功夫。分类妥善处置“僵尸企业”，全面清理取缔地下电镀、漂染以及土轧钢等环保违法企业，加快淘汰落后产能。组织商品房展示促销去库存，培育住房租赁产业，加强小产权房清理，促进房地产市场健康发展。严格执行“去杠杆”政策措施，防范化解金融风险。落实国家及省各项税费减免政策，推进电力、流通等领域改革，降低企业交易、财务、物流等成本。创新投融资机制，加快补齐交通、环保生态、教育文化、医疗卫生等基础设施短板。

深化行政体制机制改革。深入推进“放管服”改革，健全政府部门权责清单制度，进一步精简行政许可事项。深化商事制度改革，健全事中事后监管体系。全面推行“一门式”“一网式”政府服务模式。加强电子政务建设，整合政务大数据，促进政府服务管理创新。推广自贸区创新经验，打造市场化法治化国际化营商环境。

深化财税金融体制改革。强化“放水养鱼”意识，落实财税扶持政策，支持实体经济发展，夯实壮大税源基础。优化财政支出结构，促产业，保民生，集中资源办实事，办能形成可持续竞争力的大事。调整理顺市区财政管理体制。继续实施“引金入揭”计划，深化金融交流合作，鼓励支持金融改革创新，千方百计解决中小微企业融资困难。争取政策性金融推进产业、城市、生态、民生事业发展，以“PPP”、产业基金、风投、创投等多种形式，依法引导民间资本、社会资本参与项目建设。加强社会信用体系建设，优化金融生态环境。

深化农村社会各项改革。积极稳妥推进农村土地经营权确权登记颁证工作，建立健全土地流转规范管理制度。深化农村集体产权制度改革，加强农村“三资”管理，盘活集体资产，提高集体资产收益率。推进农村普惠金融“村村通”，扩大政策性农业保险覆盖面，打通金融支持现代农业发展、促进社会主义新农村建设的对接通道。

（六）加强环保生态建设，竭力实现永续发展

加大污染综合整治力度。开展绿色生产大行动，改造企业生产设备，优化生产工艺流程，确保企业绿色排放，重点建设电镀、酸洗、漂染园区，实现绿色生产。落实属地责任，坚决治理农业面源污染，发展生态农业。引导广大群众增强环保生态意识，形成绿色生产生活方式。实施环保基础设施建设三年行动计划，加快全市污水处理厂、垃圾焚烧发电厂建设，启动县（市、区）整县污水处理一体化项目建设，推进农村生活垃圾处理“户收集、村集中、镇转运、县处理”的城乡全覆盖。强化环境执法，对环境违法行为“零容忍”，顶格打击。加大榕江、练江流域治理力度，强化对龙江的保护，全面实施“河长制”，全面落实“截污、清淤、修复”责任。加强海洋生态环境污染防治。落实“气十条”“土十条”，加强大气监测，加强工业废气污染整治、扬尘整治，全面淘汰黄标车；加强固体废物、重金属综合防治、土壤污染防治，加快大南海环保中心建设。

推进绿化揭阳大行动。建设森林碳汇、生态景观林带、森林进城围城和乡村绿化美化四大工程，进一步提升全市森林覆盖率。继续开展三江植绿活动，重点在三江源头植树造林，涵养水土。实施社区公园、社区绿地规划建设专项计划，探索实行规划地块绿地面积约束指标，形成嵌入式城市“绿网”，增加全市绿色生态空间。动员全社会人人植树，为城市加绿、乡村添绿，创建一批绿色企业、绿色校园、绿色村庄、绿色社区。

（七）积极发展社会事业，补齐公共服务短板

深入开展文明创建行动。全面推进文明城市、文明县城、文明镇街、文明村居“四级联创”，实施城乡环境、交通秩序、市场秩序“三大专项整治”，下大力气整治城市卫生“脏乱差”、乱搭乱建、占道经营、交通秩序混乱等突出问题，建立健全长效管理机制，促进城市净化、绿化、美化、亮化。深入开展社会主义核心价值观教育，弘扬社会正能量。广泛开展各种群众性文化活动，弘扬优秀传统文化，传承优良家风，培育健康向上文明乡风，提高全市整体文明程度。启动丁氏光禄公祠修缮工程，加强文化遗产保护利用，积极申报国家历史文化名城。完善市文化中心配套建设，充分发挥文化设施的功能作用。抓好基层综合性文化中心建设，完善基层文化设施。推进大脊岭抗战纪念公园二期项目建设和棉湖战役遗址修建工作。做好新闻出版、广播电视、社科、档案、方志、文史等工作。

加快实施教育创先工程。实施义务教育学校现代化建设工程，继续改善办学条件，加大优质学位资源供给。实施普通高中质量提升工程，促进高中学校多样化发展。重点抓好揭阳一中科技中心、文化中心建设。围绕产学一体，推动职业教育创新发展，推进中德双元制应用科技大学筹建工作，支持揭阳职业技术学院以电子商务创业学院为突破口争创高水平学院，支持潮汕职业技术学院创办全国示范性创业名校，打造一批骨干示范中职学校。积极引导社会力量参与教育发展改革，丰富优质教育资源供给。推进互联网+教育，引入第三方云服务，形成全市学校教育数字化资源网络。加强老年教育工作，丰富老年人精神生活。联合北师大、华南师大等高校，继续抓好强师工程，壮大骨干教师队伍。

全力推进卫生创强行动。全面实施卫生创强，在加快市人民医院、慈云医院、中医院改扩建工程建设的同时，落实国开行28.7亿元政策性金融资金建设市疾控中心大楼、市妇幼保健计生服务中心住院综合大楼以及一批县级、基层卫生基础设施，更新一批现代医疗设备，补齐医疗卫生领域短板。深化医疗卫生体制改革，探索城市医院与基层医院建立城市医联体，县级医院与乡镇卫生院建立紧密型县镇一体化县域医联体，推动医疗服务重心下移和诊疗资源下沉，重点加强基层和临床服务能力。着力加强医院儿科、产科、老年病科建设，规划建设市传染病院和精神病院。实施互联网+医疗，以市级影像信息中心建设为切入，建立“医疗云”，发展智慧医疗。统筹医疗卫生与养老服务资源布局，实现资源共享、医养结合。鼓励社会资本举办医疗机构，大力发展健康服务产业。适应“全面二孩”政策需求，改进计划生育服务工作，提高出生人口素质。探索卫生系统机构编制和人事制度改革，创造条件引进高层次医疗卫生人才，加强医德医风建设，培养和造就一支医术精湛、医德高尚的医疗队伍。坚决打击“医闹”，维护正常医疗秩序。

着力加强社会保障工作。实施更加积极的就业政策，发挥公共就业服务平台作用，加强农村劳动力转移就业培训，支持技能晋升培训，促进劳动者自主就业。落实高校毕业生就业促进和创业引领计划，建设双创平台，带动青年就业创业。加强社保扩面征缴，探索城乡居民与城镇职工养老、医疗保险制度一体化，稳步提高低保等重点人群保障标准。

全面加强社会治理工作。落实《中共中央国务院关于推进安全生产领域改革发展的意见》，强化安全生产责任落实，狠抓安全隐患排查整改，重点加强“三小场所”、消防、道路交通、建筑施工等领域安全生产管理，杜绝重特大安全生产事故发生。加强食品药品安全和打假打私工作，规范市场经济秩序。创新完善立体化社会治安防控体系，保持严打高压态势，集中力量铁腕整治涉毒问题，依法严厉打击黑恶势力、“两抢一盗”、电信诈骗、涉金融领域犯罪等各类违法犯罪行为。推进社会治理“网格化”责任制，落实病残吸毒人员收治和社区戒毒社区康复工作，夯实禁毒工作基础。实施“七五”普法，完善“一村（社区）一法律顾问”工作机制和法律援助制度，抓好基层信访维稳工作，切实保障群众合法权益。同时，做好气象、地震、国防动员、民兵预备役、双拥、人防、应急、民族宗教、妇女儿童、红十字等工作。

切实办好十件民生实事。一是改扩建市区高中1所、初中3所、小学3所，新建特殊教育学校1所，新增学位6000个；落实县（市、区）主体责任，规划建设全市中小学生上下学公共交通运行体系，确保中小学生上下学交通安全。二是投入15亿元，建设市人民医院、慈云医院、中医院等28项卫生创强扩改项目，引进医疗卫生人才1000名，配置先进医疗卫生设备1000台（套）。三是加快推进引韩供水、引龙供水两大工程，加快村村通自来水工程建设，保障群众饮用水安全。四是启动智慧揭阳建设，搭建揭阳政务大数据中心和一门式政务、产业、民生服务平台，推出智慧揭阳手机APP，为企业和群众办事提供网络通道和“掌上平台”。五是全面推广农贸市场食用农产品快速检测工作及家禽“集中屠宰、冷链配送、生鲜上市”，保障人民群众舌尖上的安全。六是在全市基层新建20个体育场地设施示范点，推进体育设施进社区、进农村、进企业，全面实施全民健身工程。七是实施市区道路畅通工程，改造提升一批交通节点，配置一批指示牌、电子警察、视频监控设备等交通管控设施，推动社区、街区规划设定停车场（位）。八是实施“互联网＋精准扶贫”工程，免费为万名贫困户开展电商培训，支持千户贫困家庭创立网店。九是联合德国工商大会、德国巴伐利亚州教育集团创立双元制培训中心，创建揭阳“工匠工场”。十是建成运营锡场、里湖等9家污水处理厂，新增污水处理能力13.5万吨/天；建成运营揭阳市区及普宁生活垃圾环保处理中心，新增垃圾处理能力1800吨/天；启动市区东风河、大港河、曲溪河等5条黑臭水体治理工程，实施全市农村污水处理工程。

（八）强化政府自身建设，提高依法履职效能

加强能力建设。适应经济发展新常态，加强各级政府机关及工作人员思想建设和素质能力建设。深入学习贯彻习近平总书记系列重要讲话精神，进一步增强政治意识、大局意识、核心意识、看齐意识，特别是核心意识和看齐意识，坚持以贯彻落实中央、省委和市委精神为前提谋划推进工作。强化担当实干精神，真正把心思用在干事业上，把精力放到抓落实上，做到紧盯不放、一抓到底，全力以赴完成既定目标任务。强化改革创新能力，坚持用改革创新的思路和办法谋划发展、推动工作、解决问题。强化朴素的人民情怀，牢固宗旨意识，把人民群众对美好生活的向往作为我们最大的追求。建立政府部门行政问责、行政过错责任追究制度，发挥审计监督作用，确保各项政策措施和重点工作落到实处。

加强法治建设。强化学法用法意识，将法治思维和法治方式贯穿工作全过程。坚持“法无授权不可为、法定职责必须为”，推进政府及其部门职责法定化。加强政府立法，深入推进政务公开，规范行政行为，推进法治政府建设。自觉接受市人大及其常委会的法律监督和市政协的民主监督，广泛听取民主党派、工商联和无党派人士意见，健全决策咨询论证制度，提高科学民主依法决策水平。

加强作风建设。严格落实中央八项规定精神、国务院“约法三章”要求，巩固拓展党的群众路线教育实践活动、“三严三实”专题教育和“两学一做”学习教育成果，完善作风建设长效机制。全面落实党风廉政建设责任制，坚持问题导向、严字当头，严肃惩治行政“不作为、慢作为、乱作为”，依法查处各类违纪违法案件，坚决纠正损害群众利益的不正之风。坚持厉行节约，注重节俭办事，努力降低行政成本，以良好政风取信于民。

各位代表！时代赋予重任，人民寄予期待，实干成就梦想。让我们更加紧密团结在以习近平同志为核心的党中央周围，在省委省政府和市委的坚强领导下，不忘初心，继续前进，脚踏实地，真抓实干，弘扬愚公移山精神，全力推动经济社会健康快速发展，以优异的成绩迎接党的十九大和省第十二次党代会胜利召开
